# Pericardial tamponade during pembrolizumab treatment in a patient with advanced lung adenocarcinoma: A case report and review of the literature

**DOI:** 10.1111/1759-7714.13399

**Published:** 2020-03-17

**Authors:** Kazuaki Harada, Makoto Ogasawara, Akane Shido, Akimitsu Meno, Soichiro Oda, Shota Yoshida, Sonoe Yoshida, Ayumu Yoshikawa, Ko Ebata, Satoshi Abiko, Naoki Kawagishi, Itsuki Sano, Hisashi Oda, Takuto Miyagishima

**Affiliations:** ^1^ Department of Medical Oncology Kushiro Rosai Hospital Kushiro Japan; ^2^ Department of Internal Medicine Kushiro Rosai Hospital Kushiro Japan; ^3^ Department of Cardiovascular Medicine Kushiro Kojinkai Kinen Hospital Kushiro Japan

**Keywords:** Immune checkpoint inhibitor, pembrolizumab, pericardial effusion, pericardial tamponade

## Abstract

Several studies have demonstrated increased pericardial effusion during anti‐PD‐1 immunotherapy, and treatment in patients who have developed pericardial tamponade is controversial. In this study, we describe a 63‐year‐old woman with stage IVA lung adenocarcinoma given pembrolizumab as a first‐line therapy. After four cycles of pembrolizumab treatment, the patient suddenly developed a pericardial tamponade. Although pericardial effusion was increased, her tumor lesions were reduced. After an emergency pericardiocentesis, she continued the pembrolizumab therapy without recurrent pericardial effusions for three months until the primary tumor and lymph node metastasis progressed. Nine months after the pericardiocentesis, the patient died of progressive lung cancer, but pericardial effusion did not recur throughout the treatment course. This case study suggests that pembrolizumab therapy can be continued with a strict follow‐up in some patients with pembrolizumab‐induced pericardial tamponade.

**Key points:**

• **Significant findings of the study**

Our patient developed pericardial tamponade during pembrolizumab treatment but continued pembrolizumab treatment after emergency pericardiocentesis without recurrent pericardial effusions.

• **What this study adds**

Pembrolizumab treatments may be resumed with a strict follow‐up in some patients with treatment‐related pericardial tamponade.

## Introduction

Immune‐checkpoint inhibitors (ICIs) have become standard for advanced non‐small‐cell lung cancer (NSCLC) and other malignancies. Pembrolizumab is a monoclonal antibody against anti‐programmed cell death‐1 (PD‐1) protein and has an antitumor activity in advanced NSCLC patients either alone or in combination with cytotoxic agents.^1^, ^2^, ^3^, ^4^

ICIs are associated with a unique set of immune‐related adverse events (irAEs).^5^ It is also known that pseudoprogression, which is indicated by transient increases in tumor sizes after ICI treatments, is followed by tumor regression.^6^

Several studies show temporary and sudden increases in pericardial effusion during anti‐PD‐1 immunotherapy^7^, ^8^, ^9^, ^10^, ^11^, ^12^, ^13^, ^14^ probably due to pseudoprogression or irAEs, but the mechanisms remain unclear. Whether an anti‐PD‐1 immunotherapy should be continued after pericardial effusion progression remains a controversial issue.

Here, we report the case of a lung adenocarcinoma patient who received pembrolizumab and developed a pericardial tamponade. The pericardial effusion was temporary, and the tumor lesions shrunk. After pericardiocentesis, the patient continued treatment, and no recurrent pericardial effusion was observed for three months. At this point, however, the primary tumor and lymph node metastasis had progressed.

## Case report

**Figure 1:**
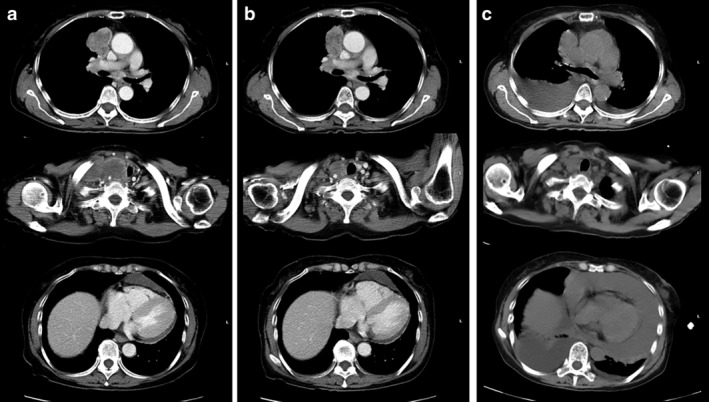
(**a**) A computed tomography (CT) scan of the chest showed a mass lesion in the right upper lobe. A right supraclavicular lymph node metastasis infiltrated into the right thyroid lobe before pembrolizumab treatment. Mild pericardial effusion was observed. (**b**) After three cycles of pembrolizumab therapy, CT scan showed a positive response in the primary lesion and lymph node metastasis. (**c**) A CT revealed a sudden increase in pericardial effusion and the tumor lesions were reduced after four cycles of pembrolizumab therapy.

A 63‐year‐old woman who was a non‐smoker presented with weight loss and right supraclavicular lymphadenopathy. Computed tomography (CT) revealed a 44 mm mass in the right upper lobe, with mediastinal lymphadenopathy of the bilateral supraclavicular and right hilar node and mild pericardial effusion. Biopsy of the right supraclavicular lymph nodes revealed adenocarcinoma. Whereas pathologic diagnosis was difficult because of low effusion, carcinomatous pericardial effusion could not be ruled out. Thus, she was diagnosed with cT2bN3M1a or stage IVA (TNM classification for lung cancer, eighth edition) lung adenocarcinoma. Molecular analyses revealed that the tumor was negative for epidermal growth factor receptor (*EGFR*) mutations and anaplastic lymphoma kinase (*ALK*) gene rearrangements and that >90% of the tumor cells expressed programmed death ligand 1 (PD‐L1). Accordingly, first‐line treatment with pembrolizumab was started at the standard dose (200 mg/kg bodyweight, triweekly). After three cycles, CT revealed a positive response in the tumor lesions (Fig 1a,b).

After four cycles, the patient presented with acute dyspnea. Her blood pressure was 94/69 mmHg, pulse rate 104/min, and oxygen saturation 93% on 3 L/minute of oxygen delivered via a mask. CT revealed massive pericardial and bilateral pleural effusions, but antitumor effects were maintained (Fig 1c). Echocardiography showed a large echo‐free space around the heart and collapse of the right ventricle, consistent with cardiac tamponade. We performed emergency pericardiocentesis and removed 900 mL of bloody effusion. Pericardial fluid analysis revealed the following: lactate dehydrogenase, 846 U/L; protein, 4.9 g/dL; glucose, 34 mg/dL; pH, 7.5; and carcinoembryonic antigen (CEA), 416.9 ng/mL. The cytological examinations revealed no malignant cells in the effusion liquid but showed numerous red blood cells, neutrophils, and very few lymphocytes.

We repeated pericardiocentesis after three days and removed 350 mL of fluid. The patient's general condition became stable. We decided to reinitiate pembrolizumab treatment. After three months, the primary tumor and lymph node metastasis had progressed, and we replaced the therapy with carboplatin plus pemetrexed. Nine months after the second pericardiocentesis, the patient died of cancer progression without any increase in pericardial effusion.

## Discussion

Table 1 lists the publications on pericardial effusion or pericardial tamponade following anti‐PD‐1 immunotherapy in lung cancer patients.^7^, ^8^, ^9^, ^10^, ^11^, ^12^, ^13^, ^14^ Increasing pericardial effusion is often observed within three months of the start of anti‐PD‐1 immunotherapy. In most cases, at least mild pericardial effusion is present before treatment and close attention is needed prior to anti‐PD‐1 immunotherapy.

**Table 1:** Comparison of case details for reports of progression of pericardial effusion following anti‐PD‐1 immunotherapy

	Pathological diagnosis	Agents	Administration cycles at presentation	Pre‐existing pericardial effusion	Interventions
Nesfeder J *et al*. 7	Adenocarcinoma	Nivo§	9	Yes	Pericardiocentesis, Pericardial window
Kushnir & Wolf^8^	SCC‡	Nivo§	5	Unknown	Pericardiocentesis, Prednisone
Shaheen *et al*. 9	Adenocarcinoma	Nivo§	1	Unknown	Prednisone
Vittorio *et al*. 10	Adenocarcinoma	Nivo§	3	Yes	Pericardiocentesis, Pericardial window
Kolla & Patel 11	SCLC†	Nivo§	weeks 9	Yes	Pericardiocentesis
	Adenocarcinoma	Nivo§	weeks 7	Yes	Pericardiocentesis, Prednisone
Yamasaki *et al*.^12^	Adenocarcinoma	Nivo§	4	Yes	Pericardiocentesis
	Adenocarcinoma	Nivo§	2	Yes	Pericardiocentesis
Tachihara *et al*.^13^	Adenocarcinoma	Pemb¶	6	No	Pericardiocentesis
Asai *et al*.^14^	Adenocarcinoma	Nivo§	1	Yes	Pericardiocentesis, Intrapericardial bleomycin

† Small‐cell carcinoma.

‡ Squamous cell carcinoma.

§ Nivolumab.

¶ Pembrolizumab.

In some cases, cytological examinations reveal no malignant cells in effusion fluids.^7^, ^8^ Shaheen *et al*. reported that pericardial effusion was effectively managed using corticosteroids,^9^ implying that their patients had irAEs. Other studies have indicated that some pericardial effusions in lung cancer patients with tamponade may be caused by pseudoprogression.^10^, ^11^, ^12^, ^13^, ^14^

In our patient, the etiology of pericardial effusion was unclear. It may have been a malignant effusion by cancer progression that was well controlled by the pericardiocentesis. However, long‐term effectiveness of pericardiocentesis is debatable. The effusion recurrence rate after simple pericardiocentesis varies (23%–55%).^15^, ^16^, ^17^ Percutaneous pericardiocentesis without extended catheter drainage has been shown to have an overall low success rate with many patients requiring further intervention for recurrences.^18^ Further, increases in malignant pericardial effusion are generally caused by cancer progression. In our patient, CT revealed a discrepancy between the rapid appearance of pericardial effusion and the antitumor effects in other lesions. Therefore, the etiology of pericardial effusion may not be simply related to cancer progression.

Fluid cytology may help to determine the etiology of the pericardial effusion during ICI treatment. Previous reports have considered a low lymphocyte count to indicate pseudoprogression.^11^, ^12^ In contrast, pericardial effusion by immune‐related serositis showed lymphocytic predominance, without evidence of malignancy.^7^, ^8^ In our patient, the pericardial fluid contained few lymphocytes without adenocarcinoma cells; however, the bloody exudative effusion had high levels of CEA, strongly suggesting a carcinomatous nature. Thus, the effusion in our patient appeared to be due to pseudoprogression rather than irAE. Moreover, we administered pembrolizumab for three months without any increase in pericardial effusion. This also supports that the etiology of the effusion is not irAE. We also ruled out infection or collagen disease from the results of the effusion analysis and her clinical course.

Continued use of anti‐PD‐1 immunotherapy is controversial after patients have recovered from cardiac tamponade. Under deteriorating irAE conditions, continued therapy does not provide a spontaneous resolution. However, if pseudoprogression is suspected and confirmed, the therapy may be continued. Monitoring pericardial effusions and other lesions is important to determine whether the cause of the effusion is pseudoprogression or not. Based on the evidence, anti‐PD‐1 immunotherapy may be resumed under close observation if the antitumor effect at other sites is notable and the pericardial fluid is controllable.^9^, ^11^, ^12^, ^13^, ^14^

In conclusion, we describe a unique case of pericardial tamponade due to pembrolizumab. We suggest that pericardial tamponade after anti‐PD‐1 immunotherapy may affect the prognosis of NSCLC patients, warranting further studies with more cases to inform appropriate management of these patients.

## Disclosure

The authors declare that they have no conflicts of interest.
